# Comparative genomics of metabolic networks of free-living and parasitic eukaryotes

**DOI:** 10.1186/1471-2164-11-217

**Published:** 2010-03-31

**Authors:** Barbara Nerima, Daniel Nilsson, Pascal Mäser

**Affiliations:** 1 Institute of Cell Biology, University of Bern, Switzerland; 2 National Livestock Resources Research Institute, Tororo, Uganda; 3 Swiss Tropical and Public Health Institute, Basel, Switzerland; 4 University of Basel, Basel, Switzerland

## Abstract

**Background:**

Obligate endoparasites often lack particular metabolic pathways as compared to free-living organisms. This phenomenon comprises anabolic as well as catabolic reactions. Presumably, the corresponding enzymes were lost in adaptation to parasitism. Here we compare the predicted core metabolic graphs of obligate endoparasites and non-parasites (free living organisms and facultative parasites) in order to analyze how the parasites' metabolic networks shrunk in the course of evolution.

**Results:**

Core metabolic graphs comprising biochemical reactions present in the presumed ancestor of parasites and non-parasites were reconstructed from the Kyoto Encyclopedia of Genes and Genomes. While the parasites' networks had fewer nodes (metabolites) and edges (reactions), other parameters such as average connectivity, network diameter and number of isolated edges were similar in parasites and non-parasites. The parasites' networks contained a higher percentage of ATP-consuming reactions and a lower percentage of NAD-requiring reactions. Control networks, shrunk to the size of the parasites' by random deletion of edges, were scale-free but exhibited smaller diameters and more isolated edges.

**Conclusions:**

The parasites' networks were smaller than those of the non-parasites regarding number of nodes or edges, but not regarding network diameters. Network integrity but not scale-freeness has acted as a selective principle during the evolutionary reduction of parasite metabolism. ATP-requiring reactions in particular have been retained in the parasites' core metabolism while NADH- or NADPH-requiring reactions were lost preferentially.

## Background

Unicellular endoparasites are the causative agents of a plethora of human diseases: malaria, sleeping sickness, Chagas' disease, toxoplasmosis, leishmaniasis, amoebic dysentery and many more, particularly in the tropics. Obligate endoparasitism among the protozoa is of polyphyletic origin. Nevertheless, the different parasites exhibit striking similarities regarding their metabolism, which is reduced - or streamlined - compared to free-living organisms. The parasites have lost metabolic functions in the course of evolution [1], presumably in adaptation to life within a foreign host organism. For instance, all obligate endoparasitic protozoa are incapable of purine de novo synthesis; they lack the corresponding genes and import exogenous purines from their hosts to synthesize nucleic acids [2,3]. The phenomenon also includes catabolic pathways: the intestinal parasites *Entamoeba histolytica *and *Giardia duodenalis *lack mitochondria, and hence oxidative phosphorylation and respiration [4]. *Trypanosoma brucei *possess mitochondria, but these are only functional in the insect stages; the bloodstream-stages rely on substrate-level phosphorylation to generate ATP [5]; the same may apply to *Plasmodium falciparum *[6]. The trend towards metabolic simplification is also apparent from endoparasitic bacteria such as *Treponema pallidum *[7] or *Mycoplasma genitalium *[8,9], which lack the genes for purine synthesis and those for pyrimidine synthesis (and the Krebs cycle as well). Thus the reduction of metabolic complexity is a convergent trait among endoparasites. Parasite metabolism is of interest not only to the evolutionary biologist but also to the pharmacologist. With the advent of large compound libraries and high-throughput screening facilities, the identification of suitable drug targets has become the bottleneck in antiparasitic hit discovery. Comparison of host and parasite metabolism may reveal vulnerable points for chemotherapeutic intervention, such as enzymes that are essential for the parasite and do not have orthologues in the host (or whose orthologues in the host are regulated differently [10]). Alternatively, prodrugs may be designed which are specifically activated by metabolic conversion in the parasite [10]. Proof of principle for this strategy was obtained with purine antimetabolites targeted towards *Toxoplasma gondii *[11].

Traditionally, the metabolism of a cell has been represented as a network of interlinked pathways [12]. More recently, representation of the metabolism as a graph, where metabolites are the nodes and enzymes are the edges (and each node appears exactly once), has provided novel insights [13]. When the mathematical concepts of graph theory that had originally been developed for quantitative analysis of social networks were applied to metabolism, the resulting graphs shared a number of characteristics with other real-world networks. Namely a short average path-length (the 'small world') and a scale-free, i.e. power-law frequency distribution of number of edges per node [14,15]. More recently, the representation of metabolic networks as hypergraphs, with different types of nodes to represent metabolites and biochemical reactions, has allowed the application of set algebra for quantitative comparison of reconstructed metabolic graphs [16]. Power-law frequency distributions in number of links are thought to result from the fact that new nodes joining the network preferentially attach to highly-linked ones ('the rich get richer' [17]). Therefore, networks are generally studied in terms of their expansion. The shrinkage of networks has mainly been analyzed in the context of how the targeted removal of nodes or edges may lead to collapse. Hence there is a third reason - besides the study of convergent evolution among parasites and the quest for new drug targets - to study parasite metabolism: it provides an opportunity to study how networks shrink in a natural way that maintains functionality. Genome-scale reconstruction of metabolic networks is most advanced for bacteria [18-20], where comparative analyses have yielded novel insights into the evolution of metabolic modules and the adaptation of microorganisms to different habitats [21,22]. Host-pathogen comparisons are contributing to antimicrobial drug discovery and to a deeper understanding of metabolic adaptations in parasitism [23,24]. Thanks to the wealth of available data, a metabolic model of *E. coli *was reconstructed that integrates information on gene expression and metabolic flux [25]. This in turn has allowed the simulation of the shrinkage of metabolic networks in bacteria by randomly deleting enzymes and calculating the effects on performance based on the predicted biomass production [26]. However, it is notoriously difficult to classify bacteria as parasitic or free-living, since the versatility of species such as *Legionella *defies categorization. For eukaryotes, the distinction between obligate endoparasites, facultative parasites, and free-living organisms is more straightforward. With the completion of the genome projects for a number of obligate endoparasitic protozoa, their metabolic networks can be reconstructed *in silico *from the predicted proteomes [27-30]. Here we compare the predicted core metabolic graphs of protozoan endoparasites, their human host, and reference organisms, aiming to identify convergent trends in the reductive evolution of metabolic networks in parasites.

## Results

### *In silico *reconstruction of core metabolic networks from parasites

To allow for subsequent comparison in terms of reductive evolution of metabolic networks in parasites, we focused on metabolic pathways that must have been present in the presumed common ancestor of all the organisms included in the present study (Table 1). These pathways comprised glycolysis, gluconeogenesis, the Krebs cycle, pentose phosphate pathway, purine and pyrimidine metabolism, and amino acid metabolism - here jointly defined as 'core metabolism'. Educt-product pairs of the core metabolic pathways were obtained from the Kyoto Encyclopedia of Genes and Genomes (KEGG [31]) *Pathway *database and used to build a reference graph of 907 nodes. Species-specific enzyme lists for the core metabolism were downloaded from KEGG *Organism *via LinkDB. The obligate endoparasites considered here for analysis of their metabolic networks were *P. falciparum, T. brucei, T. cruzi, L. major, T. parva, E. cuniculi, C. hominis *and *E. histolytica*. The following species were included for comparison: the free-living *S. pombe, S. cerevisiae, D. discoideum, D. melanogaster *and *C. elegans*, the facultative parasites *E. coli *and *C. albicans*, and the mammalian host *H. sapiens *(Table 1). The species-specific metabolic networks were built by linking the species-specific enzyme lists to educt-product pairs via the corresponding reaction numbers, obtained from KEGG *Reaction*. Two metabolites were only connected in case of carbon atom transfer between them; exchange of phosphate groups, electrons, etc. were ignored, as recommended by several authors [27,32]. Thus the current metabolites such as ATP or NADH did not become hubs, rendering the networks physiologically more meaningful. Finally, the graphs were supplemented with educt-product pairs of spontaneous, non-enzyme-catalyzed reactions followed by removal of all pairs of unconnected nodes (i.e. all the isolated edges). Additional file 1 contains the organism-specific enzyme lists by EC number, and KEGG numbers for educt-product pairs, reactions, and pathway maps.

**Table 1:** Species overview

Organism	Classification	Type	# Proteins	# Enzymes
*Homo sapiens*	Metazoa	F	25,798	324
*Escherichia coli*	Bacteria	FP	4,149	322
*Candida albicans*	Fungi	FP	14,629	299
*Saccharomyces cerevisiae*	Fungi	F	5,880	252
*Schizosaccharomyces pombe*	Fungi	F	5,003	233
*Dictyostelium discoideum*	Amoebozoa	F	13,437	228
*Drosophila melanogaster*	Metazoa	F	14,023	217
*Caenorhabditis elegans*	Metazoa	F	20,185	214
*Trypanosoma cruzi*	Euglenozoa	OP	19,607	171
*Leishmania major*	Euglenozoa	OP	8,265	163
*Trypanosoma brucei*	Euglenozoa	OP	8,712	146
*Plasmodium falciparum*	Apicomplexa	OP	5,262	139
*Entamoeba histolytica*	Amoebozoa	OP	8,162	115
*Theileria parva*	Apicomplexa	OP	4,061	97
*Cryptosporidium hominis*	Apicomplexa	OP	3,885	69
*Encephalitozoon cuniculi*	Fungi	OP	1,996	62

Species included in the study, their scientific classification, predicted numbers of protein-coding genes (according to the KEGG Genes database), and the numbers of enzymes (KEGG Pathway database) used for reconstruction of core metabolic networks (OP, obligate endoparasite; F, free-living; FP, facultative parasite).

#### Quantitative comparison of the core metabolic networks

**Figure 1:**
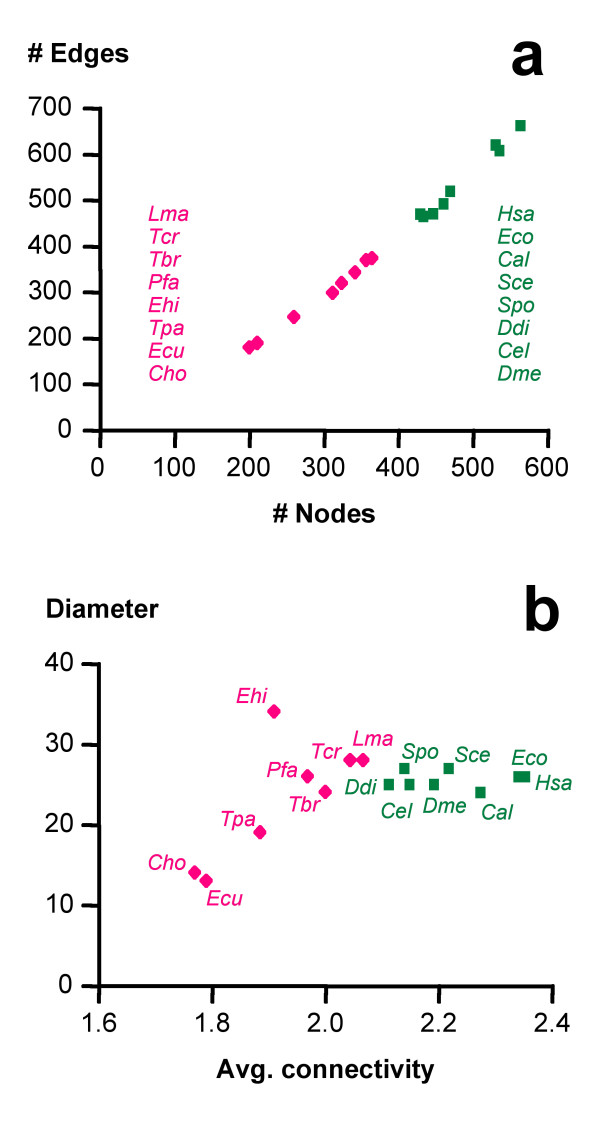
**Core metabolic graphs: parasites vs. non-parasites**. Graph properties of the predicted metabolic networks from parasites (red) and non-parasites (green). Cho, *Cryptosporidium hominis*; Ecu, *Encephalitozoon cuniculi*; Ehi, *Entamoeba histolytica*; Lma, *Leishmania major*; Pfa, *Plasmodium falciparum*; Tbr, *Trypanosoma brucei*; Tcr, *Trypanosoma cruzi*; Tpa, *Theileria parva*; Cal, *Candida albicans*; Cel, *Caenorhabditis elegans*; Ddi, *Dictyostelium discoideum*; Dme, *Drosophila melanogaster*; Eco, *Escherichia coli*; Hsa, *Homo sapiens*; Sce, *Saccharomyces cerevisiae*; Spo, *Schizosaccharomyces pombe*.

Quantitative graph properties were calculated with the programs BioLayout and GraphCrunch (Table 2). The predicted core metabolic graphs were, as expected, significantly smaller for the obligate endoparasites than for the other organisms (Figure 1a), both in average number of nodes (287 vs. 483; two-tailed Mann-Whitney test p < 0.0001) and edges (278 vs. 539; p < 0.0001). However, the parasites' graphs exhibited significantly (p = 0.0006, two-tailed Mann-Whitney test) higher densities (Table 2). The facultative parasites *E. coli *and *C. albicans *grouped together with the free-living eukaryotes (Figure 1), here collectively referred to as 'non-parasites'. When the network size was measured in terms of its diameter, i.e. the longest of all direct paths between any two nodes of the core metabolic graph as defined here, there was no significant difference between parasites and free-living eukaryotes (Table 2). The variance among network diameters and average path length was much larger for the parasites than for the non-parasites (Figure 1b, Table 2). While the nodes of the parasites' networks exhibited smaller average and maximal connectivities (Table 2; Figure 1b), there was no significant difference between the metabolic graphs of parasites and non-parasites considering the numbers of isolated edges (Table 2).

**Table 2:** Basic graph properties

	Reference	Non-parasites	Parasites	Random
Nodes	876	483 ± 52	287 ± 67	333 ± 8.6
Edges	1184	539 ± 79	278 ± 80	279 ± 7.0
Density	0.0031	0.0046 ± 0.0003	0.0070 ± 0.001	0.0049 ± 0.0002
Avg. Connectivity	2.7	2.2 ± 0.1	1.9 ± 0.1	1.7 ± 0.0
Max. Connectivity	18	11.3 ± 1.5	8.3 ± 0.7	7.2 ± 1.5
Avg. Path length	4.7	2.9 ± 0.44	1.2 ± 0.89	0.12 ± 0.07
Max. Path length (diameter)	24	25.6 ± 1.1	22.7 ± 7.1	15.4 ± 3.9
Global clustering coefficient	0.079	0.059 ± 0.009	0.039 ± 0.014	0.015 ± 0.006
Isolated edges	17	50 ± 9	51 ± 4	74 ± 6

Basic graph parameters (± standard deviation) of the reconstructed core metabolic networks, the randomly shrunk networks, and the reference network of all core metabolic reactions. Network density is the proportion of observed to total possible links, connectivity is the number of links per node, path length refers to the shortest distance connecting two nodes, and the global clustering coefficient is the number of closed triplets (triangles) over the total number of triplets.

**Figure 2:**
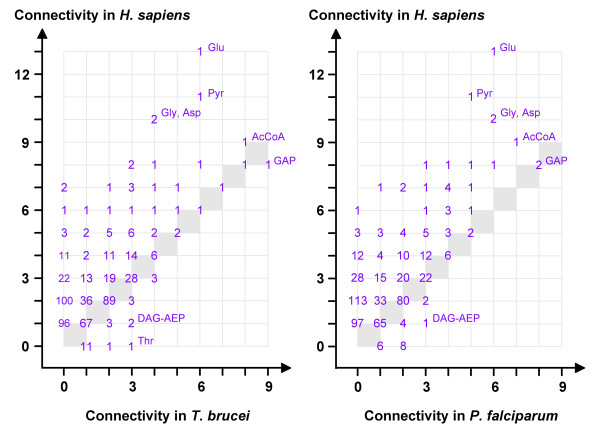
**Comparative connectivities of core metabolites**. For every degree (connectivity), the number of nodes (metabolites) in *T. brucei *(left) or *P. falciparum *(right) is plotted against the number of nodes in *H. sapiens*. The grey diagonal indicates metabolites of equal connectivity in host and parasite (AcCoA, acetyl-coenzyme A; DAG-AEP, diacylglyceryl-2-aminoethylphosphonate; GAP, glyceraldehyde-3-phosphate; Pyr, pyruvate).

The most highly connected nodes in the reconstructed network of *H. sapiens *were pyruvate and acetyl-CoA, and the amino acids glutamate, glycine and aspartate (Figure 2), which is similar to the situation in prokaryotes [33]. In parasites such as *T. brucei *or *P. falciparum*, the glycolytic intermediate glyceraldehyde-3-phosphate was the node of highest connectivity and not amino acids (Figure 2). This is in agreement with the loss of amino acid metabolic pathways in these parasites [34,35]. The biosynthetic pathways for lysine, tyrosine and tryptophan were absent in all the obligate endoparasites included in this study (in addition to their well documented inability to synthesize purines [2]). Interestingly though, a unique, possibly secreted phenylalanine hydroxylase was recently reported from *Toxoplasma gondii *[36]. Metabolites that are more highly linked in the networks of parasites than in those of their host are of pharmacological interest since structurally related antimetabolites may be more toxic to the parasite than to the host (the most detrimental attacks against networks being those directed towards hubs). However, very few metabolites exhibited a higher connectivity in the networks from parasites than in the human host (lower right corners in Figure 2). The largest difference was observed for threonine in *T. brucei*, followed by diacylglyceryl-2-aminoethylphosphonate in both *T. brucei *and *P. falciparum*. Other metabolites of higher connectivity in *P. falciparum *than in *H. sapiens *included cadaverine, homocysteine, and the fatty acid precursors phosphatidylcholine, diacylglycerol and ethanolaminephosphate. The finding that threonine was more highly linked in *T. brucei *than in *H. sapiens *is in agreement with the fact that exogenous threonine is rapidly metabolized to acetate and subsequently used for lipid synthesis in *T. brucei *[37,38].

#### How did the parasites' core metabolic networks shrink?

To study the selective forces that governed the loss of particular metabolic enzymes and pathways in obligate endoparasites, their reconstructed core metabolic graphs were compared to experimental graphs, generated by random removal of edges from the reference network until they reached the average size of the parasites' graphs. The resulting networks are unlikely to be functional and only served as a negative control. These negative control networks differed from the natural networks in that they were less coherent: the randomly shrunk graphs contained significantly (Kruskal-Wallis followed by Dunn's multiple comparison test, p = 0.0001) more isolated edges than those of either parasites or non-parasites (Table 2), and even after removal of these isolated edges, nodes of degree 1 were overrepresented in the random graphs (Figure 2, intersection with ordinate). The randomly shrunk graphs also exhibited a lower global clustering than the real metabolic networks (Table 2). The reference, the parasite, and the non-parasite graphs, while strongly differing in number of nodes and edges, all had a diameter of around 24, which is in agreement with previous studies on *E. coli *[27]. In contrast, the randomly shrunk graphs had a significantly (Kruskal-Wallis followed by Dunn's multiple comparison test, p = 0.0024) smaller diameter of around 15 (Table 2), reflecting the decomposition of the network into medium-sized entities. This was also apparent from very small average path length of the randomly shrunk graphs (Table 2).

To further identify potential factors that shaped core metabolism in the parasites, we tested the hypothesis that the frequency distribution of the number of links per node may have exerted a selective pressure: possibly, scale-freeness had to be maintained in order to preserve the robustness of the metabolic networks.

**Figure 3:**
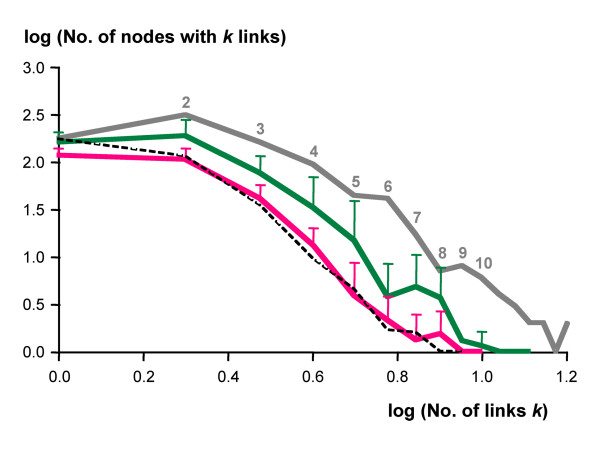
**Frequency distributions of connectivities**. Double-logarithmic plot of the frequency distributions of connectivities by node (metabolite). The parasites are shown in red (n = 8), the non-parasites in green (n = 8), error bars indicate standard deviation. Grey line, reference network; dashed line, average of randomly shrunk networks (n = 10).

The parasites and non-parasites exhibited roughly parallel, nearly scale-free frequency distributions of links per node, provided the nodes of connectivity 1 were ignored (Figure 3). The same applied to the reference network (Figure 3). This effect was caused only in part by the preceding removal of all isolated edges; also without that trimming step, the power law frequency distribution of the metabolic graphs only applied to degree 2 or more (not shown). This makes biological sense since in a perfect power law distribution the metabolic network would be dominated by dead-ended nodes of degree 1. The randomly shrunk graphs exhibited a frequency distribution of number of links almost coincident with that of the parasites' (except for the intercept with the ordinate; Figure 3). Thus scale-freeness is itself a robust trait that cannot be abolished by random deletion of edges and therefore, it cannot have acted as a selective force on metabolic networks.

**Figure 4:**
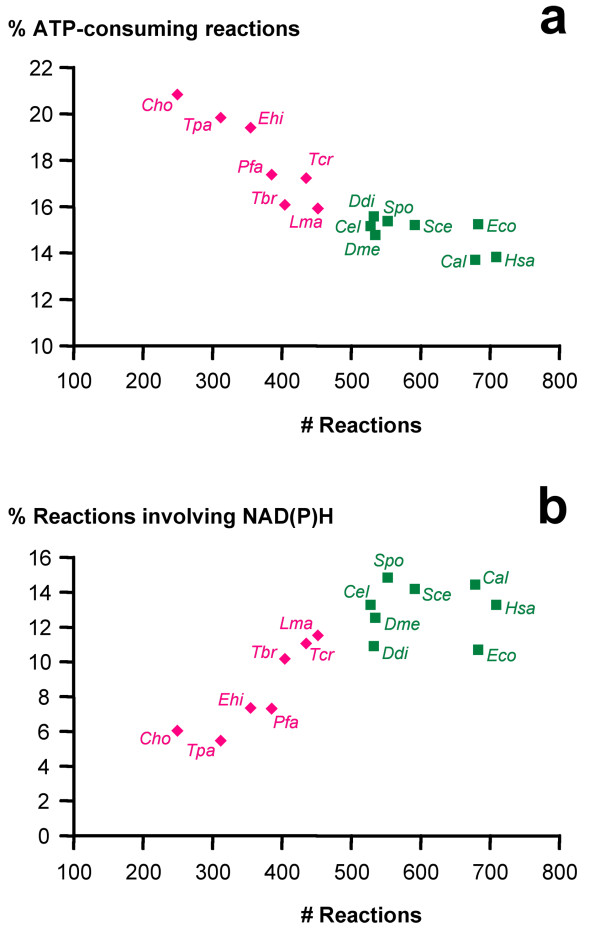
**ATP and NAD consuming reactions**. The percentage of reactions that utilize ATP (a), respectively NADH or NADPH (b), plotted against core metabolic network size. Abbreviations are the same as in Figure 1.

Another testable hypothesis was, that during the course of evolution the parasites preferentially lost enzymes catalyzing ATP-requiring reactions for economical reasons. That would be in agreement with the fact that all obligate endoparasites have lost purine de novo synthesis, which is an energetically costly pathway (for instance, it requires more ATP than pyrimidine de novo synthesis, which was lost in fewer parasites). Contrary to expectation though, the parasites' core-metabolism contained a significantly higher percentage of ATP-requiring enzymes compared to the non-parasites (two-tailed Mann-Whitney test, p = 0.0004). Those parasites with the smallest metabolic graphs in terms of number of nodes or edges, *E. cuniculi *and *C. hominis*, had the highest percentages of ATP-consuming reactions (Figure 4a). In fact, the percentage of ATP-consuming reactions in a given organism negatively correlated with that organism's total number of core metabolic reactions (Spearman coefficient -0.95, p < 0.0001), indicating that ATP-consuming reactions have a higher propensity to be retained in the course of network evolution. The opposite trend was observed for NADH and NADPH (Figure 4b). Note that as current metabolites, ATP, NADH and NADPH themselves did not represent hubs in the present graphs.

## Discussion and Conclusion

To study in an evolutionary context the convergence in reduction of metabolic complexity among different parasites, we focused on metabolic pathways that must have been present in the free-living ancestor of all the organisms included (Table 1). Core metabolic graphs comprising glycolysis, gluconeogenesis, the Krebs cycle, pentose phosphate pathway, purine, pyrimidine, and amino acid metabolism were reconstructed from the KEGG collection of databases for obligate endoparasitic eukaryotes (referred to as 'parasites'), and free-living or facultative parasitic organisms (referred to as 'non-parasites'). As expected, the resulting core metabolic graphs of the parasites were significantly smaller than those of the non-parasites when regarding the number of nodes (metabolites) or edges (reactions). The validity of this finding depends on the status of functional annotation in the KEGG data: the non-parasites predominantly constituting model organisms, the observed difference in network size may be caused by the higher accuracy of annotation in the predicted proteomes of the non-parasites compared to the parasites. However, the finding that the parasites possess reduced core metabolic networks is in agreement with other studies [24] and with biochemical data [1]. Furthermore, the facultative pathogen *Candida albicans *(which is not a model organism) clustered with the free-living eukaryotes, while the obligate endoparasite *Trypanosoma brucei *(which has a high quality of gene annotation) clustered with the parasites (Figure 1). Thus we do not think that the striking differences in network size between the parasites and the non-parasites were artefacts due to the different standards of functional annotation in the analyzed proteomes. The parasites' networks did not exhibit smaller diameters than those of the non-parasites (Figure 1b) while control graphs, shrunk to the size of the parasites' by random deletion of edges from the core metabolic reference graph, were fragmented and had significantly smaller diameters (Table 2). The total number of reactions in a given organism negatively correlated with the percentage of ATP-consuming reactions (Figure 4a) and positively correlated with the percentage of NADH- or NADPH-utilizing reactions (Figure 4b). This indicates that ATP-requiring enzymes, respectively the genes encoding their catalysts, have a higher propensity to be retained in the course of network evolution and that the retention of ATP-requiring reactions may be one of the selective forces acting on network evolution. A possible interpretation could be that ATP-consuming reactions are more likely to be essential than reactions that do not involve ATP, so loss of the corresponding enzymes would be more likely to be harmful. We tested this hypothesis on the results from the *Saccharomyces *Genome Deletion Project [39-41] and found an interesting accordance: of all *S. cerevisiae *genes annotated with EC number (n = 518), 18% were essential for growth on rich glucose medium. Of the genes encoding enzymes that catalyze ATP-consuming reactions (n = 87), 22% were essential while for reactions involving NADH or NADPH (n = 87), the fraction of essential genes was 14%. However, the differences were not statistically significant (p = 0.27, two-tailed Fisher's exact test). In summary, focusing on core pathways for the reconstruction of metabolic graphs has permitted comparative genomics between obligate endoparasites and free-living (or facultative parasitic) eukaryotes and has identified the preferred retention of ATP-consuming reactions, and the enhanced loss of NADH- or NADPH- utilizing reactions, as potential selective forces which may have acted during the reductive evolution of parasitic metabolism.

## Methods

For all of the selected core metabolic pathways, educt-product pairs of reactions and the corresponding enzymes were retrieved manually from the Reference pathway maps of the KEGG database [42] and supplemented with data kindly provided by H. Ma and A.P. Zeng (German Research Center for Biotechnology, Braunschweig, Germany). Organism-specific enzyme lists were also obtained from KEGG, via LinkDB. The networks were reconstructed as directed graphs, but for all subsequent analyses treated as undirected. The basic network properties such as average connectivity or network diameter were determined with BioLayout [43], GraphCrunch [44], and Bioperl v1.5.2. The metabolites' connectivities and the connectivities' frequency distribution were determined with self-developed Perl scripts. Shrinkage of networks was performed with a Perl script that randomly removed educt-product pairs from metabolic maps until a given size was reached. These scripts are available upon request.

## Authors' contributions

BN performed the reconstruction of metabolic networks, their comparative analysis, and drafted the manuscript. DN calculated the quantitative network parameters. PM conceived the study, performed the statistical analysis, and finalized the manuscript. All authors read and approved the final manuscript.

## Supplementary Material

Additional file 1 **Reconstructed core metabolic networks in tabular form**. MS Excel file with one spreadsheet per organism, containing predicted enzymes (EC number), corresponding educts, products, reaction, and pathway maps (KEGG numbers). Click here for file
